# Missing the context: The challenge of social inequalities to school‐based mental health interventions

**DOI:** 10.1002/jcv2.12165

**Published:** 2023-04-28

**Authors:** Karen L. Mansfield, Obioha C. Ukoumunne, Sarah‐Jayne Blakemore, Jesus Montero‐Marin, Sarah Byford, Tamsin Ford, Willem Kuyken

**Affiliations:** ^1^ Department of Psychiatry University of Oxford Oxford UK; ^2^ NIHR ARC South West Peninsula Department of Health and Community Sciences Faculty of Health and Life Sciences University of Exeter Exeter UK; ^3^ Department of Psychology University of Cambridge Cambridge UK; ^4^ UCL Institute of Cognitive Neuroscience London UK; ^5^ Teaching, Research & Innovation Unit Parc Sanitari Sant Joan de Déu Sant Boi de Llobregat Spain; ^6^ Consortium for Biomedical Research in Epidemiology & Public Health (CIBER Epidemiology and Public Health ‐ CIBERESP) Madrid Spain; ^7^ King's College London King's Health Economics Institute of Psychiatry, Psychology and Neuroscience London UK; ^8^ Department of Psychiatry University of Cambridge Cambridge UK

## Abstract

Given well‐established links between socio‐economic adversity and mental health, it is unsurprising that young people's mental health is deteriorating amidst economic crises. The World Health Organisation (WHO) recognises mental health as “crucial to personal, community, and socio‐economic development” and outlines goals to reshape environments such as schools to protect mental health. Schools offer an ideal setting to promote wellbeing and prevent mental ill‐health during a key developmental window. We describe how social inequalities present a challenge to designing school‐based interventions for prevention and promotion for mental health and wellbeing, and suggest priorities to aid and evaluate their effectiveness.

## Graphical abstract

The effectiveness of universal school‐based mental health interventions is significantly challenged by increasing contextual inequalities. This editorial perspective suggests priorities to make school‐based interventions more inclusive and engaging, co‐designed with students, tailored to schools, addressing multiple determinants, to reduce inequalities whilst promoting wellbeing.

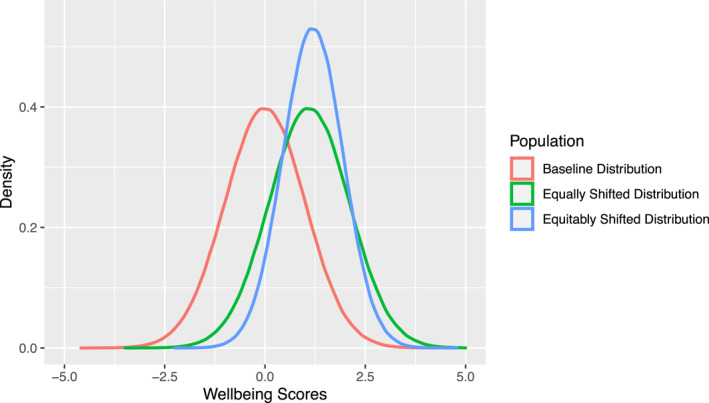

**Key points**

- There is growing scepticism regarding the effectiveness of universal school‐based mental health interventions.
- Most school‐based mental health interventions fail to adequately address the significant impact of contextual inequalities on young people's health and wellbeing, missing vulnerable students.
- We present an analysis of the challenges posed by inequalities, and suggest priorities that could help reduce inequalities whilst promoting wellbeing, in interventions co‐designed with students.
- Priorities include current and representative data and to assess needs, tailoring to schools, addressing multiple determinants together, and maximising inclusivity, engagement and agency.

## CHALLENGES

The recently published null results of a large‐scale randomised controlled trial (MYRIAD, My Resilience In Adolescence), assessing the effectiveness of school‐based mindfulness training (SBMT) on mental health (Kuyken et al., 2022), have raised scepticism regarding SBMT and universal school‐based mental health programmes (Cuijpers, 2022). We argue that for these programmes to be effective, we first need to understand and address the challenges posed by social inequalities. As social disparities grow, the range of needs becomes more diverse and one size becomes less likely to fit all.

### Contextual inequalities

Socio‐economic factors (e.g., housing, food‐security, community services) are significant determinants of health, and in the UK especially, inequalities have been increased by austerity and the COVID‐19 pandemic (Marmot & Allen, 2020). Students from more disadvantaged homes were already at increased risk of poor mental health during the pandemic (Mansfield et al., 2021), and increasing numbers are experiencing poverty (Iacobucci, 2023). Mental health is also closely interwoven with poor engagement and attendance at school, both exacerbated by unsupported needs (Finning et al., 2022), further enhancing inequalities. Children who are unwell, unsafe, or experiencing mental health difficulties will struggle to engage at school, whether it's with the core curriculum, social‐emotional‐learning (SEL), or other interventions.

**FIGURE 1:**
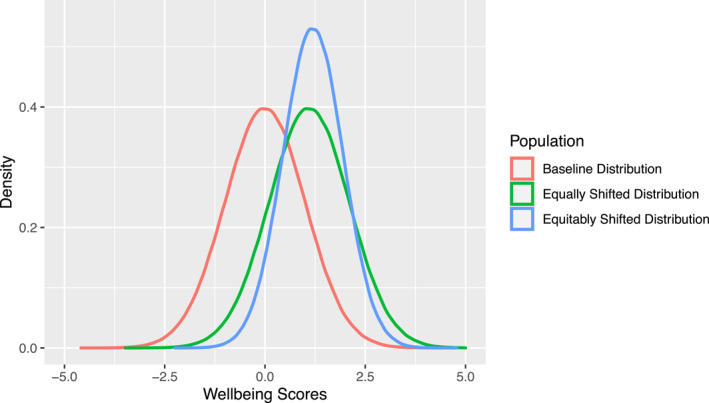
Theoretical distributions of adolescent wellbeing, with scores around the mean on the *x*‐axis and density (probabilities) on the *y*‐axis. In red is a theoretical baseline population distribution with mean = 0 and standard deviation = 1. The green line depicts the aim of a universal approach, to shift the entire distribution equally in a positive direction. The blue line depicts a theoretical outcome of a more equitable approach, with a higher mean but also a lower standard deviation, implying both improved wellbeing in the population and reduced inequalities.

This implies that the most vulnerable students are often the least likely to benefit from school‐based interventions, particularly universal interventions that assume a ‘one size fits all’ approach, with the aim to shift the complete distribution of outcomes (Figure 1, distribution in green). School‐based interventions should ideally aim to reduce inequalities (Fusar‐Poli et al., 2021), or at least ensure that they are not reinforced.

### Universal and selective approaches

Selective interventions that target those most ‘at risk’ might be more effective and cost‐effective in redressing inequalities (Hetrick et al., 2016), but bring different disadvantages. Accurately identifying those likely to develop mental health disorders can be challenging and lead to the ‘prevention paradox’, whereby many of those not receiving the intervention later develop the conditions the intervention aimed to prevent (Rose, 1985). Also, targeting those ‘at risk’ can induce stigma (Gronholm et al., 2018), and might increase perceived inequalities, which independently predict mental health over objective inequalities (Piera Pi‐Sunyer et al., 2022). Combining universal and selective approaches has been suggested, with uncertainty over how to make this more effective than a single approach (Fusar‐Poli et al., 2021).

## PRIORITIES

With greater inequalities, the distribution of social determinants (e.g. access to leisure activities) will have a larger standard deviation, and the distribution of wellbeing will likely reflect this pattern. An intervention that reduces inequalities and promotes wellbeing should both improve outcomes and reduce the standard deviation (Figure 1, distribution in blue). This could be achieved by an equitable approach that draws the wellbeing of the population towards the top end of the distribution, so that those with the lowest initial wellbeing derive the largest benefits, with smaller gains for those with moderate and good wellbeing. Addressing this complex challenge involves several priorities (Table 1).

**TABLE 1:** Key challenges posed by social inequalities to the design of school‐based mental health interventions and the associated priorities proposed to overcome these challenges.

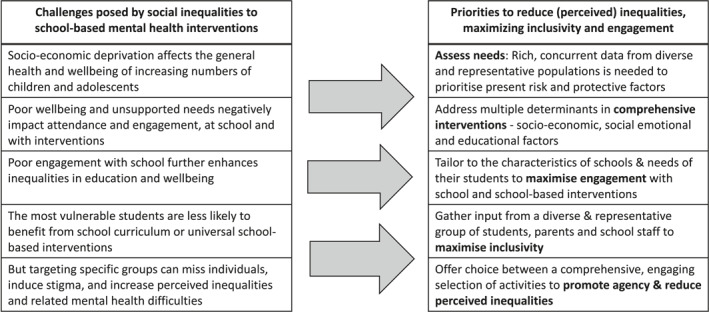

*Assessing determinants/needs*: Designing inclusive interventions requires understanding the full range of determinants in the relevant population. Population mental health data often lack up‐to date information or representative samples, which can negatively impact the design of interventions (Ford et al., 2021; Pierce et al., 2020). Research informing school‐based interventions needs to ensure that sampling is representative and that variability between schools and regions is considered.

*Comprehensive approach*: Once the full range of determinants is identified, these can best be addressed simultaneously in combined interventions, with both effectiveness and cost‐effectiveness in mind. A key example is integrated models of prevention, which simultaneously address multiple risk factors, enhance protective factors, and activate multiple mechanisms, such as the ‘PATHS to PAX’ integrated intervention (Bradshaw et al., 2020; Domitrovich et al., 2010). An integrated model that promotes wellbeing and reduces inequalities might target social determinants (e.g., healthy meals, sports) and school engagement, as well as SEL components (e.g., mindfulness, drama).

*Maximising inclusivity*: Ensuring that all students in each school have the opportunity to engage with school‐based interventions depends on their engagement with school itself. A combination of administrative data held by schools, surveys, and qualitative research with students, parents and school staff can be used to understand the range of needs and the extent to which these are already met by existing school provision. Where possible, interventions should tailor to the unmet needs expressed by students, including those who struggle with attendance and engagement.

*Maximising engagement/agency*: A diverse and inclusive group of students can help to identify a comprehensive and engaging selection of components and modes of implementation that fit within the broader goals of the intervention. Offering a choice between these components rather than allocating students prescriptively also promotes agency, and avoids errors in identifying those at risk, stigma, and the reinforcement of perceived inequalities.

## ASSESSING EFFECTIVENESS

With multiple mechanisms addressed in one intervention and a choice of components, both the effectiveness of the intervention and the role of individual determinants can be difficult to assess. Methods have been proposed for assessing intervention effectiveness by measuring the area between the pre‐ and post‐intervention distribution curves, which estimates the proportion of the distribution that has benefited from the intervention, accounting for multiple causality effects (Sarkadi et al., 2014). Additionally, complier average causal effect estimation can provide an estimate of intervention effectiveness with optimal implementation, and for participants who comply with the intervention, as demonstrated for the PATHS to PAX integrated model (Bradshaw et al., 2020). Untangling the potentially moderating and mediating roles for different determinants could provide insight into the contributions of a broad range of factors to adolescent wellbeing.

## CONCLUSION

Guidelines set out by the WHO (2022) and challenges highlighted by learnings from large scale trials suggest that school‐based mental health interventions need a reset, to ensure they are inclusive, engaging and reduce inequalities. Key priorities to achieve this are to address multiple determinants, tailor to the school context, and maximise engagement inclusively, aided by co‐production with students, parents, and school staff.

## AUTHOR CONTRIBUTIONS

Karen L. Mansfield: Conceptualization; Writing – original draft; Writing – review & editing. Obioha C. Ukoumunne: Funding acquisition; Writing – review & editing. Sarah‐Jayne Blakemore: Funding acquisition; Writing – review & editing. Jesus Montero‐Marin: Writing – review & editing. Sarah Byford: Funding acquisition; Writing – review & editing. Tamsin Ford: Funding acquisition; Writing – review & editing. Willem Kuyken: Conceptualization; Funding acquisition; Supervision; Writing – original draft; Writing – review & editing.

## CONFLICTS OF INTEREST STATEMENT

Karen L. Mansfield, Obioha C. Ukoumunne, Sarah‐Jayne Blakemore, Jesus Montero‐Marin, Sarah Byford and Tamsin Ford declare no conflict of interest related to this editorial. Willem Kuyken is the Director of the Oxford Mindfulness Centre and receives royalties for several books on mindfulness.

## ETHICS STATEMENT

No ethical approval was required for this editorial perspective.

## Data Availability

Data sharing is not applicable as no data analyses are reported in this editorial perspective. However, data from the MYRIAD trial are available upon reasonable request to Willem Kuyken (willem.kuyken@psych.ox.ac.uk), subject to an approved proposal and a signed data access agreement.
